# In vivo validation of the functional role of MicroRNA-4638-3p in breast cancer bone metastasis

**DOI:** 10.1007/s00432-023-05601-5

**Published:** 2024-02-01

**Authors:** R. L. Akshaya, I. Saranya, G. Margaret Salomi, P. Shanthi, R. Ilangovan, P. Venkataraman, N. Selvamurugan

**Affiliations:** 1 Department of Biotechnology, School of Bioengineering, SRM Institute of Science and Technology, Kattankulathur, Tamil Nadu 603 203 India; 2 SRM-DBT Platform, SRM Institute of Science and Technology, Kattankulathur, Tamil Nadu 603 203 India; 3 Department of Pathology, Dr. A.L.M. PG Institute of Basic Medical Sciences, University of Madras, Taramani, Chennai, Tamil Nadu India; 4 Department of Endocrinology, Dr. A.L.M. PG Institute of Basic Medical Sciences, University of Madras, Taramani, Chennai, Tamil Nadu India; 5 Department of Medical Research, Faculty of Medicine and Health Sciences, SRM Institute of Science and Technology, Kattankulathur, India

**Keywords:** Breast cancer, Bone metastasis, miR-4638-3p, ATF3, Caudal artery injection

## Abstract

**Purpose:**

Skeletal metastases are increasingly reported in metastatic triple-negative breast cancer (BC) patients. We previously reported that TGF-β1 sustains activating transcription factor 3(ATF3) expression and is required for cell proliferation, invasion, and bone metastasis genes. Increasing studies suggest the critical regulatory function of microRNAs (miRNAs) in governing BC pathogenesis. TGF-β1 downregulated the expression of miR-4638-3p, which targets ATF3 in human BC cells (MDA-MB-231). In the present study, we aimed to identify the functional role of miR-4638-3p in BC bone metastasis by the caudal artery injection of the MDA-MB-231 cells overexpressing mir-4638 in the mice.

**Methods:**

MDA-MB-231 cells overexpressing miR-4638 were prepared by stable transfections. Reverse transcriptase quantitative PCR was carried out to determine the expression of endogenous miR-4638-3p and bone resorption marker genes. X-ray, micro-CT, and Hematoxylin & Eosin studies were used to determine osteolytic lesions, trabecular structure, bone mineral density, and micrometastasis of cells.

**Results:**

The mice injected with MDA-MB-231 cells overexpressing miR-4638-3p decreased the expression of bone resorption marker genes, compared to MDA-MB-231 cells injection. Reduced osteolytic lesions and restored bone density by MDA-MB-231 cells overexpressing miR-4638-3p were observed. Similarly, the mice injected with MDA-MB-231 cells overexpressing miR-4638-3p showed a better microarchitecture of the trabecular network. A few abnormal cells seen in the femur of MDA-MB-231 cells-injected mice were not found in MDA-MB-231 cells overexpressing miR-4638.

**Conclusion:**

The identified functional role of ATF3 targeting miR-4638-3p in BC bone metastasis in vivo suggests its candidature as BC therapeutics in the future.

## Introduction

Breast cancer (BC) accounts for one-third of cancer diagnoses in women and remains the cardinal cause of mortality globally among adults and middle-aged females. Since the mid-2000s, there has been a steady surge in the BC incidence rates (about 0.5% every year), primarily attributed to decreasing fertility rates and an increase in obesity (Seigel et al. 2022). Specifically, in the case of solid tumors, secondary metastasis remains to be the causal factor for mortality (Dillekås et al. 2019). Oligometastasis to bone or combined with other organs remains to be most frequently diagnosed (~ 70%) in metastatic BC patients (Liu et al. 2020). The tumor-secreted factors are critical in forming and priming the premetastatic niche that favors the survival and growth of metastasized tumor cells in the bone (Chiou et al. 2021). Intrinsic factors, including cytokines, microvesicles, nucleic acids, etc., secreted by tumors or in response to the cancer were identified to modulate the bone microenvironment and govern tumor progression (Ubellacker and McAllister 2016; Li et al. 2023).

The bone matrix serves as an enriched reserve of latent TGF-β1, and tumor-induced osteoclastic resorption of bone results in an acidic environment ideal for activating TGF-β1 (Hering et al. 2001). TGF-β1 is a stimulatory cytokine extensively studied for its role in BC invasion and bone metastasis (Macroni et al. 2019). Activating transcription factor 3 (ATF3), an adaptive and stress response gene, is reported to be dysregulated in human cancers. An increased expression of ATF3 in human BC was found to be due to its localization within the most frequently amplified region, chromosome 1q amplicon in BC (Yin et al. 2008). A study suggested that TGF-β1 could induce the expression of ATF3, and the induced ATF3 upregulated the expression of TGF-β1 in MCF-1OCA1a cells, thus forming a positive feedback loop for TGF-β signaling. Further, upregulated ATF3 was observed to be vital for TGF-β1 to increase the expression of its Epithelial–Mesenchymal Transition (EMT)-related genes, including snail, slug, and twist, hence increasing BC cell motility (Yin et al. 2010; Ku et al. 2020; Yan et al. 2021). Cell proliferation (cyclin A1), invasion (matrix metalloproteinase 13; MMP13), and metastasis (Runx2) genes were found to be ATF3 target genes (Kwok et al. 2009; Gokulnath et al. 2017; Rohini et al. 2019, 2021). Thus, targeting ATF3 might aid in controlling BC progression and subsequent metastasis (Huang et al. 2021).

In recent years, there are reports suggesting the potential of non-coding RNAs (miRNAs and circRNAs) as cancer biomarkers (Shenglong Li 2021; Bevacqua et al. 2022; Guo et al. 2023). Increasing studies suggest the critical regulatory function of microRNAs (miRNAs) in governing BC pathogenesis (Bertoli et al. 2015; Bhat et al. 2019; Akshaya et al. 2023). miRNAs are post-transcriptional regulators that regulate gene expression under physiological and pathological conditions (O'Brien et al. 2018). miRNAs such as miR-155, miR-125b, miR-21, and miR-222 were clinically associated with tumor resistance to standard treatments, thus serving as predictors of response to BC therapeutics (Campos-Parra et al. 2017). Overexpression of miR-183-5p and miR-492 promoted proliferation and invasion and induced pre-neoplastic phenotypes in the 3D culture of BC cells, recapitulating the phenotypes observed upon the loss of connexin 43 (Cx43), a tumor suppressor gene. Although both miRNAs did not directly target Cx43, they disrupted the epithelial polarity in BC cells via downregulating gap junctional and various other cell junction gene targets (Chasampalioti et al. 2019; Naser Al Deen et al. 2022).

Previously, we reported on the tumor suppressive role of miR-4638-3p in controlling TGF-β1 stimulated BC progression and bone metastasis in vitro*.* Overexpression of this miRNA reduced proliferation, invasion, and migration, promoted apoptosis, and arrested human BC cells (MDA-MB-231) at G0/G1 phase (Akshaya et al. 2022). One of the primary concerns associated with the cell culture-based study is the reproducibility of the results in vivo. The inability of the in vitro models to provide information on the heterogeneity of cancer, its microenvironment, and associated stromal cells has impeded the understanding of tumor pathogenesis, treatment response, and systemic response to the drug (Sajjad et al. 2021). Nude mice models are prevalently used to decipher the molecular mechanism behind tumor progression and metastasis (Park et al. 2018). Traditionally, the intracardiac injection was the best approach for studying the bone metastasis of BC cells. However, alternative models were suggested due to the increased mortality and other vital organ metastases (Campbell et al. 2021). Caudal artery injection of cells to study bone metastases is recently developed and proven to be the more appropriate model for investigating bone metastasis that has reduced mortality, decreased rates of vital organ metastases, and preferential delivery of cells to the bones of hind limbs (Farhoodi et al. 2020). In this study, we aimed to validate the functional role of miR-4638-3p in influencing the bone metastasis of human BC cells in vivo using a caudal artery injection model system. Our results suggested that the stable overexpression of miR-4638-3p could reduce the bone metastatic potential of human BC cells, highlighting the candidature of this miRNA as a therapeutic agent for treating BC bone metastasis.

## Materials and methods

### Transformation and restriction digestion

The pCMV-MIR (empty vector; pCMV-EV; Cat #PCMVMIR, Origene, USA) and pCMV-MIR4638 (mir-4638 overexpressing vector; pCMV-miR; Cat # SC401776, Origene, USA) were transformed into JM-109 cells by CaCl_2_-mediated transformation as mentioned in (Asif et al. 2017). The transformed cells were then selected in an LB agar plate using kanamycin (25 μg/ml) to obtain individual colonies, which were then cultured overnight in LB broth. Respective plasmids were then isolated using Qiagen Miniprep (Qiagen, Valencia, CA) and subjected to XhoI digestion to verify their size.

### Stable cells generation and selection

MDA-MB-231 cells were transfected with pCMV-EV or pCMV-miR using lipofectamine 2000. Post 48 h of transfection, stably transfected cells were selected using increasing concentrations of neomycin sulfate, starting from 50 μg/ml to 200 μg/ml (till ~ 90% of cells died). The survived cells were then selected using clonal rings and were propagated individually to form clones. Six clones were chosen for MDA-EV (MDA-MB-231 cells transfected with empty vector) and MDA-miR (MDA-MB-231 cells transfected with pCMV-MIR4638) (C1 to C6) and propagated individually. The propagated cells were then harvested, and the most appropriate clone was selected by determining the expression of miR-4638-3p using reverse transcriptase quantitative polymerase chain reaction (RT-qPCR) analysis (Akshaya et al. 2022).

### Bone metastasis model system

The Centre for Cellular and Molecular Biology, Hyderabad, India, provided the female nude mice utilized in the studies. The mice were 4 to 5 weeks old and weighed about 20–25 g. According to the Institutional Guidelines and Regulations for the Care and Use of Laboratory Animals established by the Committee for the Purpose of Control and Supervision of Experiments on Animals (CPCSEA) in accordance with the Prevention of Cruelty to Animals Act 1960, Government of India, all experimental procedures were carried out under these guidelines. The Institutional Animal Ethical Committee of SRM IST, located in Kattankulathur, India, granted permission for the study (IAEC No. SAF/IAEC/280622/024). Three groups of animals (n = 8) were used: control (1 × PBS), MDA-EV-, and MDA-miR-injected groups. Following anesthesia (IP administration of ketamine (100 mg/kg) and xylazine (10 mg/kg), 1 × PBS (100 μl) or respective cells resuspended in 1 × PBS (~ 60,000 cells/100 μl) were injected into the caudal artery as mentioned previously (Han et al. 2020; Zhong et al. 2020). After being observed for 30 days post-injection, the animals were sacrificed by anesthetic overdose. For further studies, the femur and tibiae were dissected and fixed for 48 h at room temperature in 10% neutral buffered formalin.

### Reverse transcriptase quantitative polymerase chain reaction (RT-qPCR)

Total RNA was isolated from the cells, quantified, and subjected to complementary DNA (cDNA) synthesis using miScript PCR assay (Qiagen, CA). Further, quantitative PCR was performed using miScript primer assay (Qiagen, CA) in Quant Studio 3 applied biosystem. The fold change in the expression of miR-4638-3p was calculated using ∆∆CT method. RPL13A/B was an endogenous control (Akshaya et al. 2022).

Fresh bones collected from respective groups were grounded into powder using liquid nitrogen, and total RNA was isolated. The cDNA was then synthesized using an iScript cDNA synthesis kit (Biorad, USA), and the qPCR analysis was performed with TB green premix Ex Taq II (Takara, USA) using the primers for Trap5 and Cathepsin K (Table 1). The relative expression of bone resorption marker genes (Trap5 and Cathepsin K) was estimated using the ∆∆CT method and normalized with RPL13A/B (Rohini et al. 2018; Malavika et al. 2020).

**Table 1:** List of primers used for bone resorption gens in qPCR analysis

SI. NO	Name	Primer (5′-3′)
1	m-TRAP5	F: CCAACCTGGCTTCTCTGACTTA
		R: AAGAGAGAAAGTCAAGGGAGTGGC
2	m-Cathepsin K	F:GCAGATGGGCAGATGTTTGTG
		R:ATACCTGGGAATGAACTGGTCG

### X-ray and µ-CT analyses

A set of collected and formalin-fixed bones was subjected to X-ray analysis (X-ray tube voltage: 58 kV and exposure time: 1.2 to 1.6 s), and bone density was calculated using ImageJ software. Further, these bones were subjected to µ-CT imaging using SKYSCAN, Bruker, USA. The dissected femur and tibia bones were imaged using the following parameters: 9 μm pixel size, 55 kV of X-ray tube voltage, 197–198 μA of X-ray tube current, 360º rotation with 0.5 degrees per scan, and a partial width of 100% (Kim et al. 2021; Shim et al. 2022). A total area of 10 mm (5 mm above and below the femur and tibia joint) was fixed for scanning. Reconstruction, analysis, and modeling were performed using NRecon, CTAn, and CTVox software, respectively.

### Histological staining

Post-fixation, the samples were decalcified using 20% EDTA solution (pH adjusted to 7.4) at 4º C for 14 days. The decalcification solution was changed every 72 h. The decalcified bones were washed with 1 × PBS twice and stored at 4ºC until further analyses. The decalcified samples were then paraffin embedded, longitudinally sectioned (4 µm), and subjected to hematoxylin & eosin (H&E) staining, as described previously (Sun et al. 2018).

### Statistical analysis

All the quantitative data were generated using biological triplicates (n = 3 samples). One-way ANOVA analysis was used to determine the significance of the data, and mean ± standard deviations were used to depict the values; *p*-value < 0.05 was deemed statistically significant. Eight nude mice per group were utilized in animal experiments, which were determined using Cochran’s sample size determination formula (Z = 1.96; p = 0.1 (10% mortality); d = 0.12 (12% precision)).

## Results

### Generation of MDA-MB-231 cells stably overexpressing miR-4638-3p

**Fig. 1:**
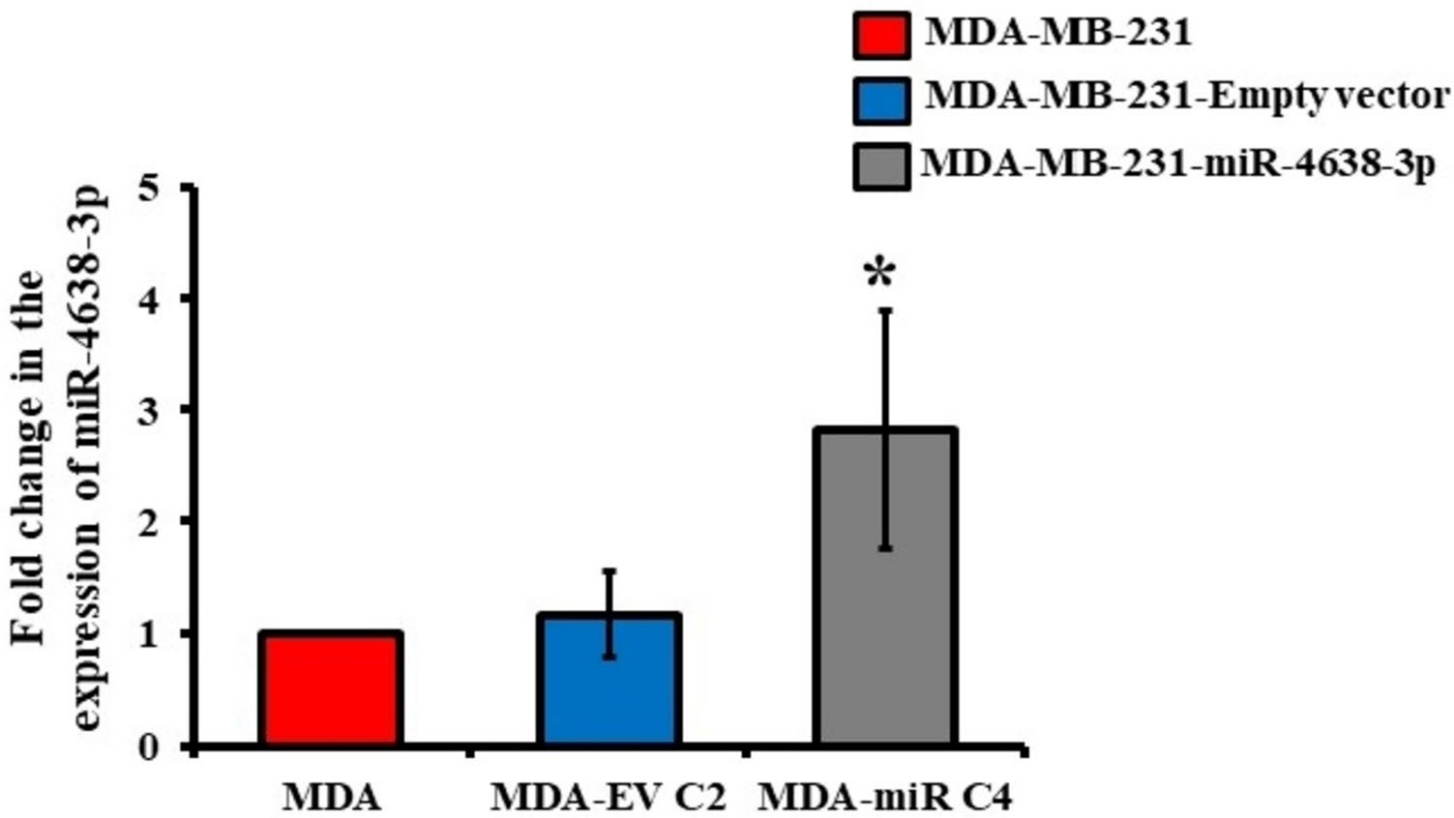
Generation of MDA-MB-231 cells stably overexpressing miR-4638-3p. Total RNA was isolated from MDA-MB-231 cells (MDA), stable MDA-MB-231 cells overexpressing empty vector (MDA-EV C2), and MDA-MB-231 cells overexpressing miR-4638 (MDA-miR C4) cells. The isolated RNA was subjected to cDNA synthesis, followed by qPCR analysis with primers for miR-4638-3p. * a substantial increase compared to parental MDA and stable MDA-EV C2 cells

To validate the function of miR-4638-3p in vivo, six clones from MDA-MB-231 cells stably transfected with pCMV or pCMV-miR were picked up for each of these stable cells. The most appropriate clone was selected by determining the expression profile of miR-4638-3p using RT-qPCR analysis. Results indicated a significant upregulation in the expression of miR-4638-3p in clone 4 (C4) of MDA-miR when compared to parental MDA-MB-231 cells (MDA), suggesting successful stable transfection and processing of overexpressed precursor mir-4638 into miR-4638-3p in these cells (Fig. 1). In case of MDA-EV cells, clone 2 (C2) was selected as the relative expression of miR-4638-3p in these cells was not significantly different, compared to parental MDA cells (Fig. 1). Together, this result indicated the successful generation of MDA-EV and MDA-miR stable cells.

### Determination of the functional role of miR-4638-3p in vivo by RT-qPCR analysis

**Fig. 2:**
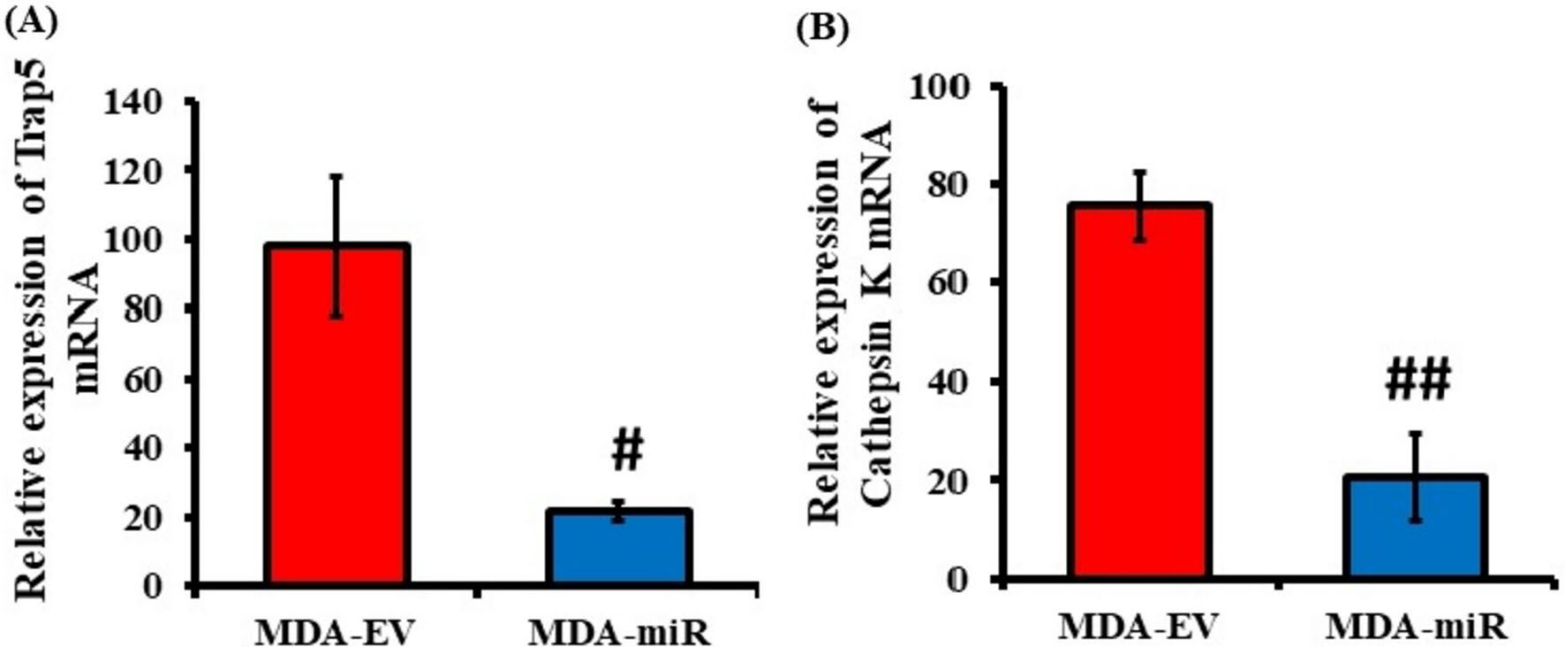
Expression of bone resorption marker genes in MDA-EV- and MDA-miR-injected mice. Thirty days after the injection of the MDA-EV or MDA-miR cells into mice, they were sacrificed, and a set of fresh bones was used for total RNA isolation, cDNA synthesis, and followed qPCR analysis using the primers for Trap5, Cathepsin K, and RPL13AB. The relative expression of bone resorption marker genes. **A** Trap5 and **B** Cathepsin K was determined. RPL13AB was used for the normalization. # depicts a substantial decrease relative to the MDA-EV group (*p* < 0.01). ## depicts a substantial reduction relative to the MDA-EV group (*p* < 0.005)

The stable cells (MDA-EV and MDA-miR) generated were injected into nude mice via caudal artery injection in the tail, as described in the methodology. Thirty days post-injection, the animals were sacrificed, and femur and tibia bones were collected. A set of fresh bones from each group were ground into powder using liquid nitrogen and mixed with RNAiso plus reagent. Total RNA was extracted and subjected to RT-qPCR analysis to determine the expression of bone resorption marker genes such as Trap5 and Cathepsin K. The results showed a significant reduction in the mRNA expression of Trap5 and Cathepsin K in the bones of MDA-miR-injected mice when compared to MDA-EV-injected mice (Fig. 2). These results suggest that the stable overexpression of miR-4638-3p could impair the bone resorption potential of MDA-MB-231 cells in vivo.

### X-ray analysis of bone metastasis in vivo

**Fig. 3:**
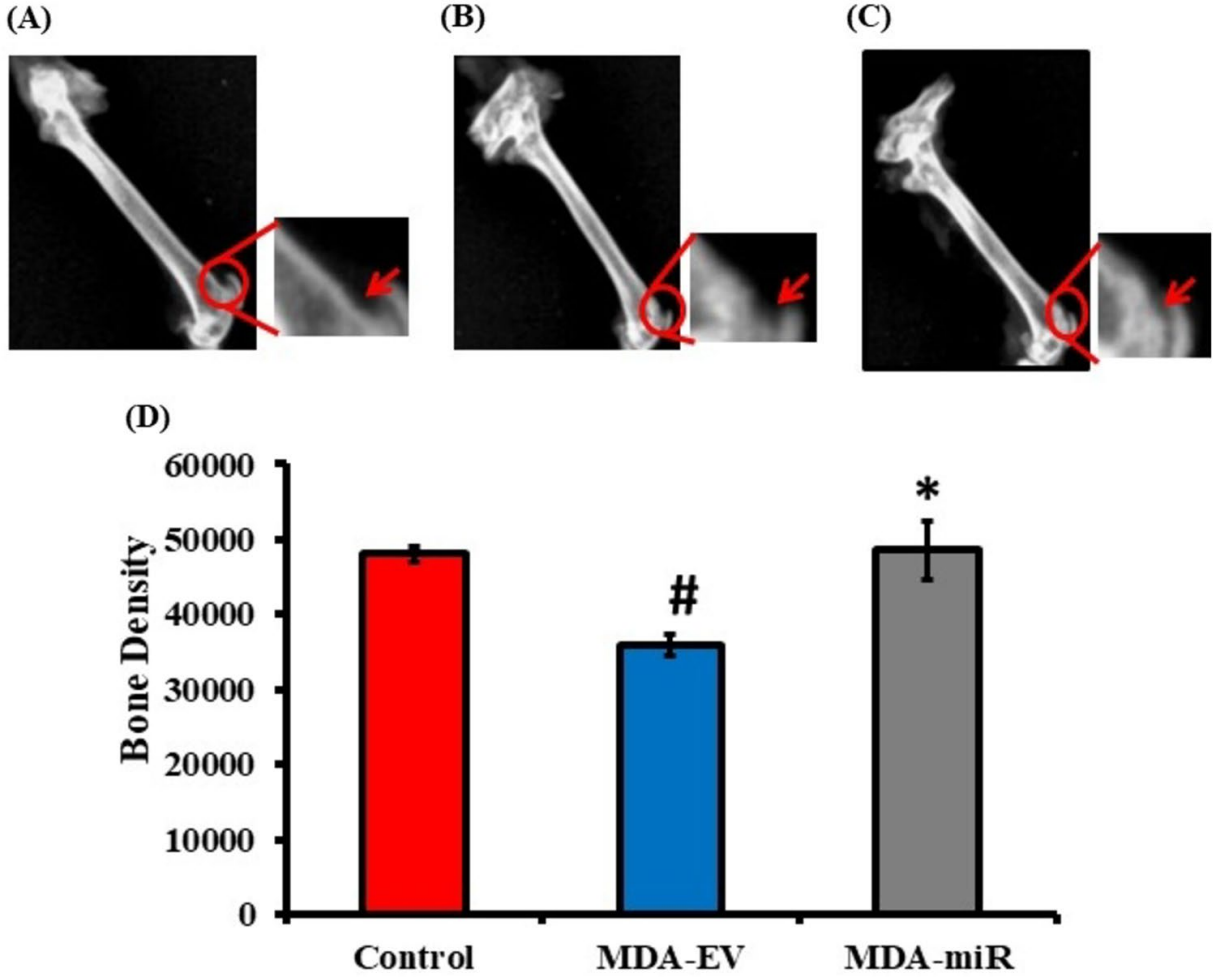
X-ray analyses of bone metastases after injection of MDA-EV and MDA-miR cells into mice. The mice were injected with 1 × PBS (control- 100 µl), MDA-EV cells, or MDA-miR cells (60,000 cells/100 µl) via caudal artery injection. Thirty days after injection, mice were sacrificed, and femur and tibia bones were collected. The collected bones were subjected to X-ray analyses. **A** Control, **B** MDA-EV cells injected, and **C** MDA-miR cells injected. **D** The region with an osteolytic lesion (marked as a circle in red) was selected, and bone density was calculated using ImageJ software. # indicates a substantial decrease compared to the control (*p* < 0.001); * indicates a substantial increase compared to MDA-EV-injected group (*p* < 0.005)

The femur and tibia bones obtained were fixed using 10% buffered formalin for 48 h and stored at 4º C until further analysis. The fixed bones were then subjected to X-ray analysis, as mentioned in the methodology. The results showed a presence of a mild osteolytic lesion in the femurs of the mice injected with MDA-EV cells (Fig. 3B). In contrast, there was a decreased osteolytic lesion when the mice were injected with MDA-miR cells (Fig. 3C). The mice injected with 1 × PBS alone as control group showed no osteolytic lesions in the femurs (Fig. 3A). The obtained X-rays were quantified (marked as circle in red) using ImageJ software, and bone density was calculated. A significant reduction in bone density was observed in the mice injected with MDA-EV cells compared to control. When the mice were injected with MDA-miR, the reduction of bone density found in MDA-EV cells was rescued (Fig. 3D), suggesting the role of miR-4638-3p in controlling bone metastasis in vivo.

### µ-CT analysis of bone metastasis in vivo

**Fig. 4:**
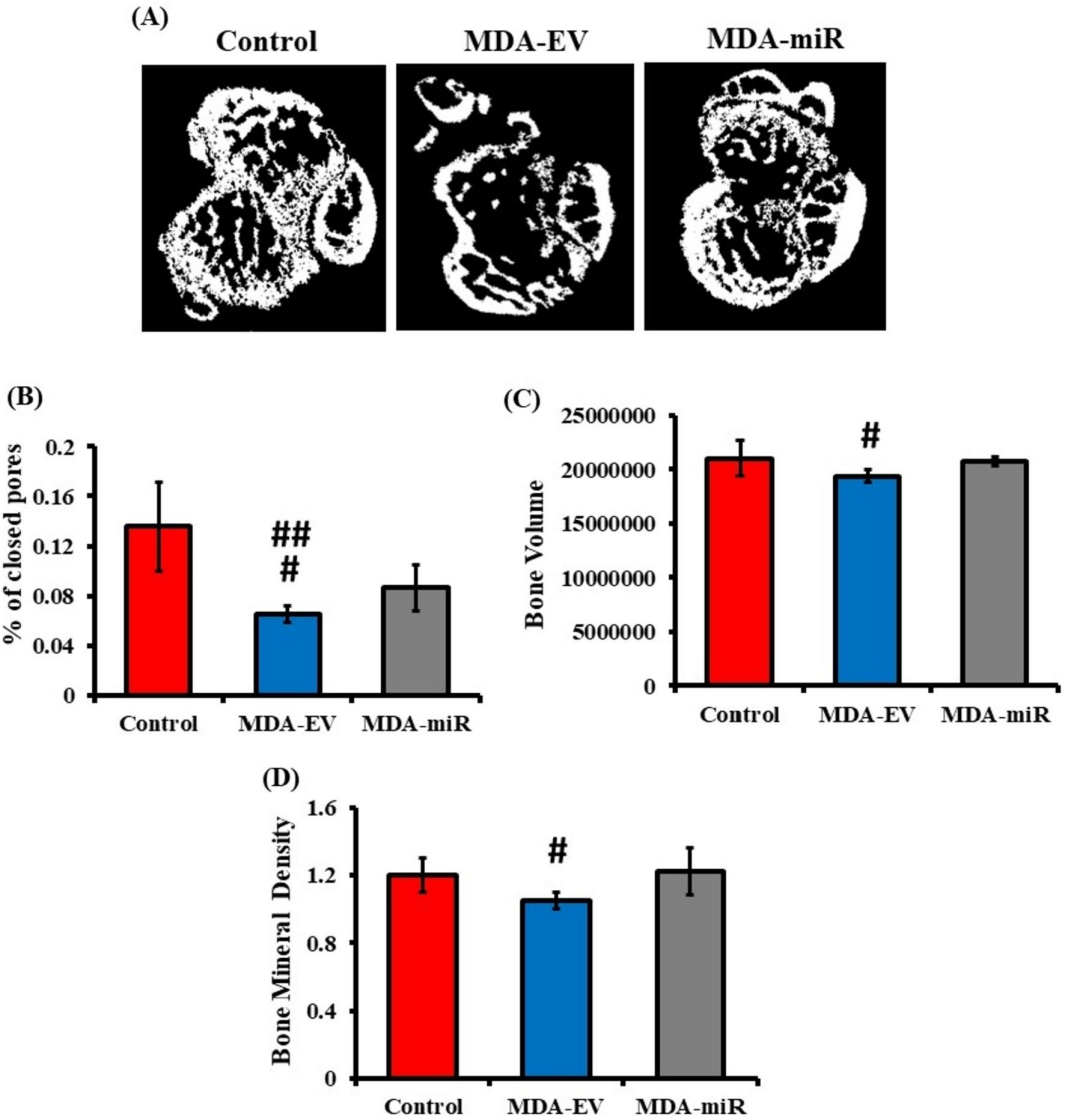
µ-CT analysis of femur and tibia bones after injection of MDA-EV and MDA-miR cells into mice. Thirty days post-injection with 1 × PBS (control), MDA-EV, or MDA-miR cells, mice were sacrificed, and femur and tibia bones were collected. They were subjected to µ-CT analysis. **A** Representative µ-CT images of femur and tibia bones (5 mm above and below the femur and tibia joint). Bar graphs depicting **B** the % of closed pores present, **C** bone volume, and** D** BMD**.** # a substantial decrease compared to control (*p* < 0.05); ## a substantial decrease compared to MDA-miR-injected group (*p* < 0.005)

The µ-CT analysis was performed to verify bone metastasis, and the quantitative data were used to determine the porosity, bone volume, and BMD. The representative images of a pixel of the analyzed bones are shown in Fig. 4A. Compared to control mice, there was notable damage and a decrease in the microarchitecture of the trabecular bones of MDA-EV-injected mice, whereas the mice injected with MDA-miR cells showed a better microarchitecture of the trabecular network compared to the mice injected with MDA-EV cells (Fig. 4A). The percentage of closed pores has an inverse correlation with the porosity of the bone. A significant reduction in the % of closed pores in MDA-EV-injected mice was observed, compared to both control and MDA-miR-injected groups, indicating increased bone porosity (Fig. 4B). In terms of bone volume and BMD, there was a significant reduction in the bone volume and BMD in the femur and tibia of MDA-EV-injected mice compared to the control. In MDA-miR-injected mice, the bone volume and BMD were closely restored compared to the control (Fig. 4C and D). Together, these results suggested that overexpression of miR-4638-3p could reduce bone metastasis in vivo.

### Histological staining

**Fig. 5:**
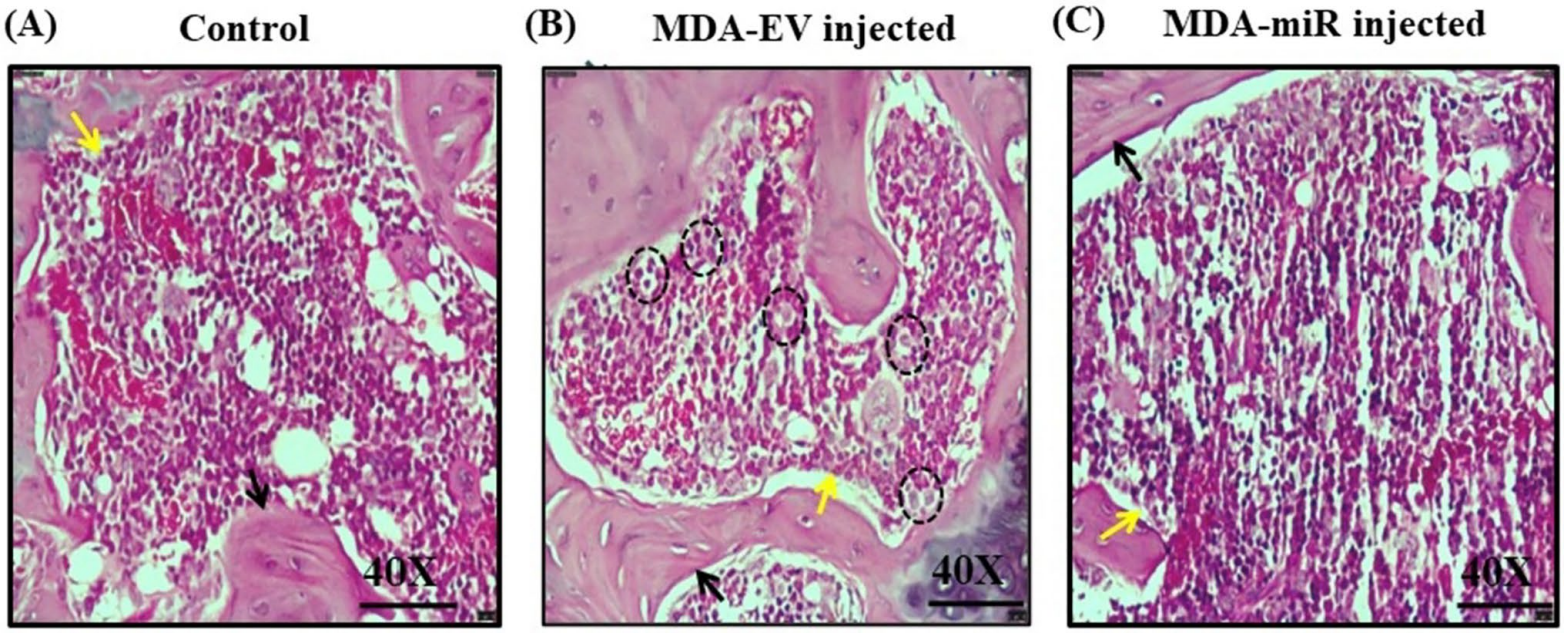
H&E staining of femur bones. Thirty days after the injection of mice with 1 × PBS (control), MDA-EV, or MDA-miR cells, they were sacrificed, and femur bones were collected. The collected femur bones were fixed with 10% formalin and decalcified for 14 days with 20% EDTA. The decalcified bones were paraffin embedded, sectioned into 4 µm slices longitudinally, and subjected to H&E staining. Representative images of H&E staining of femur bones of **A** Control, **B** MDA-EV cells-injected mice, and **C** MDA-miR cells-injected mice. Regions highlighted in dotted black circles indicate the abnormal cells detected. Black and yellow arrows indicate the bone and bone marrow, respectively

H&E staining was conducted with the femur bones. The results indicated the presence of abnormal foreign cells in the bone marrow environment of MDA-EV mice, which might be due to the micrometastasis of injected MDA-MB-231 cells (highlighted in black dotted circles) (Fig. 5B). In contrast, these cells were completely absent in the bone marrow environment of control and MDA-miR-injected mice (Fig. 5A and C). These results indicated that overexpression of miR-4638-3p could curb the bone metastasis of MDA-MB-231 cells in vivo.

## Discussion

BC is a primary concern that critically affects women's health and quality of health. Due to the tumor heterogeneity and resistance acquired to the administered therapies, there is an increasing demand for novel and effective molecules for BC treatment (Shinde et al. 2023). Recent development in bioinformatics and experimental approaches has shed light on the regulatory role of miRNAs and their candidature as next-generation therapeutic regimens. Various sophisticated pre-clinical and clinical trials are required to understand the molecular function of miRNAs and their candidature as therapeutics in vivo (Ding et al. 2020; Haider et al. 2022). Previously, we reported the role of miR-4638-3p in reducing BC proliferation, invasion, and bone metastasis by directly targeting ATF3 in vitro (Akshaya et al. 2022). In this study, we investigated the functional role of miR-4638-3p in regulating BC progression and bone metastasis in vivo. First, MDA-MB-231 cells stably overexpressing empty vector (MDA-EV) or mir-4638 (MDA-miR) were generated by stable transfection. Researchers utilize stable cell lines for various applications, such as studying differentiation, gene expression, and cellular toxicity (Tandon et al. 2018). The stable cells generated were selected, and six clones, each for MDA-EV and MDA-miR cells, were selected. The most appropriate clone of the generated stable cells was selected based on the expression of miR-4638-3p using RT-qPCR (Fig. 1).

Stable cell-mediated delivery of miRNAs into in vivo models has become increasingly significant in recent years (Wang et al. 2019; Zhou et al. 2022; Yu et al. 2023). The transient transfection generates temporary effects making it suitable for studying short-term effects of gene expression, and stable transfection is useful to develop and understand the permanent impact of genetic modifications. For instance, stable overexpression of miR-10b enhanced the expression of EMT and stemness markers in MCF-7 cells via negative regulation of PTEN and continued activation of Akt signaling (Bahena-Ocampo et al. 2016). Likewise, another study reported on the tumor suppressive role of miR-1287-5p in reducing cell growth in vitro and in vivo upon stable overexpression in BC cell lines (Schwarzenbacher et al. 2019). The stable overexpression of miR-133b reduced the BC metastasis in vivo via targeting Translocase of Inner Mitochondrial Membrane 17 homolog A (TIMM17A), a mitochondrial protein (Li et al. 2019). Overexpression of miR-3613-3p suppressed the growth and lung metastases of hBC cells in vivo (Chen et al. 2021). In a recent study, stable overexpression of miR-4521 inhibited cell proliferation, invasion, migration and EMT, and reduced DNA damage response in BC via targeting FOXM1 (Kuthethur et al. 2023).

Nude mice models are prevalently used to decipher the molecular mechanism behind tumor progression and metastasis (Park et al. 2018; Li et al. 2021). Traditionally, the intracardiac injection was the best approach for studying the bone metastasis of BC cells. However, alternative models are suggested due to the increased mortality and other vital organ metastases by cardiac injection (Campbell et al. 2021). Caudal artery injection of cells to study bone metastases is recently developed and proven to be the more appropriate model for investigating bone metastasis that has reduced mortality, decreased rates of vital organ metastases, and preferential delivery of cells to the bones of hind limbs (Farhoodi et al. 2020). Studies suggested caudal artery injection as a model for bone metastasis (Han et al. 2020; Zhong et al. 2020; Kuchimaru et al. 2021).

Our results indicated a significant reduction in the expression of Trap5 and Cathepsin K in MDA-miR-injected mice when compared to MDA-EV-injected mice (Fig. 2), suggesting that overexpression of miR-4638-3p could reduce BC-induced osteoclastogenesis in vivo. Similar to our findings, a study reported that the knockdown of miR-214-3p in nude mice reduced osteoclast activity and prevented bone metastasis of inoculated BC cells (Liu et al. 2017). Further, X-ray and µ-CT analyses were conducted to assess the bone metastasis of injected cells. Results from X-ray analysis suggested the presence of a mild osteolytic lesion in the femur of MDA-EV-injected mice (Fig. 3B) and a significantly reduced bone density in MDA-EV-injected mice when compared to control and MDA-miR-injected mice (Fig. 3D). While X-ray analysis offers details regarding the presence or absence of osteolytic lesions, it has limited resolution. It cannot generate three-dimensional, quantifiable images to measure tumor-induced bone loss (Geffre et al. 2015). At present, µ-CT analysis is extensively utilized to accurately and efficiently study bone structure and microarchitecture (Kim et al. 2021). It can provide intricate details regarding micrometastasis and the process of tumor progression, specifically highlighting the tumor-induced bone changes (Young et al. 2023). In our study, the µ-CT analysis indicated notable damage in the microarchitecture in the femur of MDA-EV-injected mice, compared to control or MDA-miR-injected mice (Fig. 4A). In contrast, the mice injected with MDA-miR cells stably overexpressing miR-4638-3p were observed to have significantly reduced BC-induced bone porosity (Fig. 4B), restored bone volume (Fig. 4C) and BMD (Fig. 4D), compared to MDA-EV-injected and control mice. Together, these results suggested that overexpression of miR-4638-3p could reduce bone metastasis in vivo.

H&E staining remains the gold standard technique used to primarily diagnose BC, followed by special staining for molecular markers is required for further subtyping (Li et al. 2018). This staining offers details about the architecture of the tissue components and aids in investigating cellular morphology, which is essential for cancer diagnosis (Shovon et al. 2022). Our results from H&E staining suggested the presence of abnormal foreign cells in the bone marrow microenvironment of the femur of MDA-EV-injected mice (Fig. 5). In contrast, no such cells were seen in control or MDA-miR-injected mice. However, these abnormal cells were observed only in one of six femur samples of MDA-EV-injected mice randomly analyzed. 

## Conclusion

In the present study, we analyzed the functional role of ATF3 targeting miR-4638-3p in BC progression and bone metastasis in vivo. Stable overexpression of miR-4638-3p in BC cells decreased the bone resorption potential, reduced osteolytic lesions, maintained the porous microarchitecture of the trabecular bones, and restored bone volume and BMD. In addition, stable overexpression of this miRNA reduced the micrometastasis of BC cells to bones. Thus, miR-4638-3p could aid in curbing the bone metastasis of BC cells, emphasizing the candidature of this miRNA as BC therapeutics in the future. However, a limiting factor of this study is the minimal effectiveness of miR-4638-3p observed in vivo, emphasizing the need for an extended and better-optimized animal study to determine the functional role of this miRNA in controlling BC bone metastasis.

## Data Availability

The data supporting the findings of this study are available from the corresponding author upon reasonable request.

## Abbreviation

BC: Breast cancer
ATF3: Activating transcription factor 3
pre-mir: Precursor microRNA
EMT: Epithelial–mesenchymal transition
CPCSEA: Committee for the purpose of control and supervision of experiments on animals
µ-CT: Microcomputed tomography
H&E: Hematoxylin & Eosin
TIMM17A: Translocase of inner mitochondrial membrane 17 homolog A
BMD: Bone mineral density
